# Oxidative balance score and dietary phytochemical index can reduce the risk of colorectal cancer in Iranian population

**DOI:** 10.1186/s12876-023-02826-z

**Published:** 2023-05-25

**Authors:** Shahrooz Bentyaghoob, Fereshteh Dehghani, Arezoo Alimohammadi, Zainab Shateri, Masoud Amini Kahrizsangi, Elham Tavassoli Nejad, Mehran Nouri, Bahram Rashidkhani

**Affiliations:** 1 Department of Clinical Nutrition, School of Nutrition and Food Sciences, Shiraz University of Medical Sciences, Shiraz, Iran; 2 Department of Nutritional Sciences, Texas Tech University, Lubbock, TX USA; 3 Department of Nutrition, School of Public Health, Iran University of Medical Sciences, Tehran, Iran; 4 Department of Food Hygiene and Quality Control, School of Nutrition and Food Sciences, Shiraz University of Medical Sciences, Shiraz, Iran; 5 Department of Nutrition and Biochemistry, School of Medicine, Ilam University of Medical Sciences, Ilam, Iran; 6 Department of Community Nutrition, School of Nutrition and Food Sciences, Isfahan University of Medical Sciences, Isfahan, Iran; 7 Department of Clinical Nutrition, School of Nutrition and Food Sciences, Isfahan University of Medical Sciences, Isfahan, Iran; 8 Department of Community Nutrition, School of Nutrition and Food Sciences, Shiraz University of Medical Sciences, Shiraz, Iran; 9 Student Research Committee, Shiraz University of Medical Sciences, Shiraz, Iran; 10 Department of Community Nutrition, Faculty of Nutrition and Food Technology, National Nutrition and Food Technology Research Institute, Shahid Beheshti University of Medical Sciences, Tehran, Iran

**Keywords:** Oxidative balance score, Dietary phytochemical index, Colorectal cancer

## Abstract

**Background:**

No previous study has assessed the association between oxidative balance score (OBS) and dietary phytochemical index (DPI) with colorectal cancer (CRC) simultaneously. Therefore, this study investigated the association between OBS and DPI with the odds of CRC among the Iranian population.

**Methods:**

This hospital-based age and sex-matched case–control study was conducted between September 2008 and January 2010 (142 controls and 71 cases were entered for analysis). New diagnosed CRC cases were selected from the Cancer Institute, Imam Khomeini Hospital of Tehran. Dietary intakes were determined by a semi-quantitative food frequency questionnaire (FFQ). Then, dietary indices were calculated by food items and nutrient intake. Logistic regression was utilized for assessing the tertiles of OBS and DPI.

**Results:**

In multivariate analysis, OBS was associated with a 77% reduction in CRC odds in the last tertile than the first tertile (odds ratio (OR) = 0.23, confidence interval (CI): 0.07–0.72, P_trend_ = 0.017). Also, we found a 64% reduction in CRC odds in the last tertile of DPI compared to the first tertile (OR = 0.36, CI: 0.15–0.86, P_trend_ = 0.015).

**Conclusions:**

A diet rich in phytochemicals and anti-oxidants, including fruits and vegetables (citrus fruits, colored berries, and dark-green leafy vegetables) and whole grains, may reduce the CRC odds.

**Supplementary Information:**

The online version contains supplementary material available at 10.1186/s12876-023-02826-z.

## Background

Colorectal cancer (CRC) is the third most common cancer worldwide [1]. CRC is a multifactorial disease with modifiable (diet, smoking, and sedentary lifestyle) and non-modifiable (age, sex, family history, and race) determinants [2, 3]. Lifestyle and diet could positively or negatively affect CRC and play a significant role in its prevention [4]. High intakes of specific vitamins (including vitamins E, D, and C) and micronutrients (Ca, Mg, Zn, and Se) have been shown to protect against CRC. In contrast, alcohol consumption, high iron intake, and smoking exacerbate this condition [5].

It has been found that oxidative stress, an imbalance between pro-oxidant and anti-oxidant status, plays a key role in CRC pathogenesis [2, 6]. Oxidative stress induces macromolecular (protein, lipids, and deoxyribonucleic acid (DNA)) damage [7], which can subsequently provoke mutagenesis and carcinogenesis [8, 9]. Therefore, oxidative balance score (OBS) has been introduced to indicate the overall exposure balance of pro-oxidant and anti-oxidant [10]. A higher score of OBS indicates a higher exposure to anti-oxidants than pro-oxidants [5]. Previous studies have demonstrated an inverse association between OBS and CRC [11, 12]. To our knowledge, no study has been conducted on the relationship between this index and the risk of CRC in the Middle-Eastern population. Dietary intake of the Middle-Eastern population has its unique pattern: large portion sizes with high consumption of refined grains (bread and white rice) and a greater percentage of energy from carbohydrates [13].

Moreover, epidemiological studies have shown the protective effects of fruit and vegetable-rich diets against CRC, mainly attributed to their phytochemical content [14]. Phytochemicals are known as non-nutritive bioactive compounds (including phenolic compounds, isoprenoids, and organosulfur compounds) [15] with anti-cancer properties, which affect cancer initiation, promotion, and progression through anti-oxidant properties, anti-inflammatory activities, and regulation of cellular signaling pathways [16]. The phytochemical load of a diet is obtained by the dietary phytochemical index (DPI), calculated as the percentage of daily energy intake derived from phytochemical-rich foods [17]. A reverse association between DPI score and various diseases has been shown in previous studies, including obesity [18, 19], insulin resistance [15], stroke [20], knee osteoarthritis [21], and breast cancer [16, 22]. To our knowledge, studies have not yet simultaneously demonstrated the effect of OBS and DPI on CRC odds. Therefore, this study investigated the association between OBS and DPI with the odds of CRC among Iranian population.

## Methods

### Study population

**Fig. 1:**
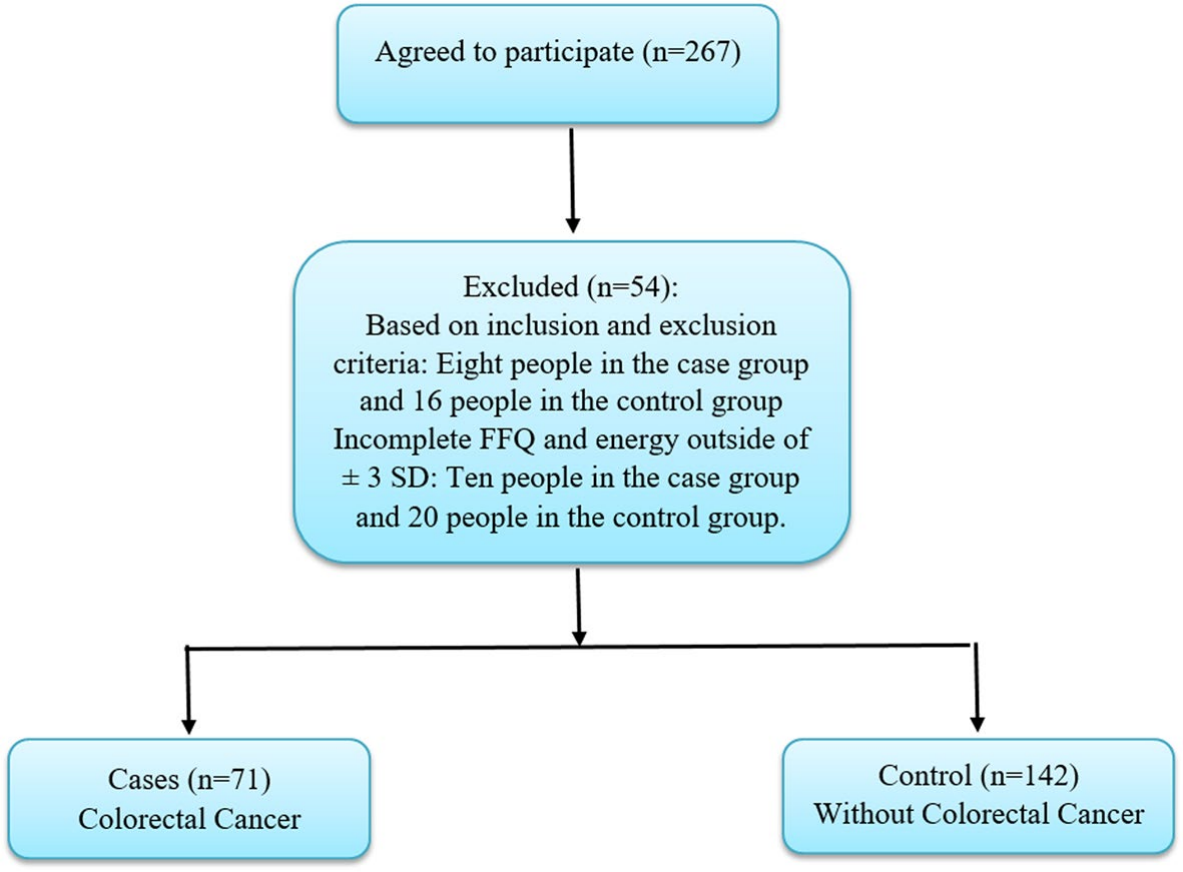
Flow chart of the study

This hospital-based study was done at the Cancer Institute, Imam Khomeini Hospital of Tehran. Our study was conducted between September 2008 and January 2010. The sample size was calculated based on the previous study [23], considering the odds ratio (OR) = 0.45, α = 0.05, β = 0.2. The patients who had no previous diagnosis of cancer more than six months prior to the interview were included. The participants were between 40–75 years old and had no diagnosis of cancer elsewhere or a family history of adenomatous polyposis. Patients with acute and non-neoplastic diseases admitted to the same hospital were chosen as controls. Each case was matched for age (five-year classifications) and sex with two controls. At first, 267 patients were selected (178 controls and 89 cases). Fifty-four patients were removed from the study due to unwillingness, total energy intake (out of mean ± 3 standard deviations (SDs)), and incomplete food frequency questionnaire (FFQ) (Fig. 1). Finally, 142 controls and 71 cases were entered into the analysis. Some details of this study have been published previously [24].

### Dietary assessment

Dietary intakes were determined by a semi-quantitative FFQ. The validity of FFQ has been assessed among Iranian adults [25]. In this interview, a valid food album [26] for the convenience of participants was used. After calculating the gram of foods, the Nutritionist IV (version 7.0; N-Squared Computing, Salem, OR, USA) was utilized to calculate energy and intake of nutrients.

### Dietary indices

Based on a method by Goodman et al. [27], the OBS was determined by collecting data from four classes: dietary pro-oxidants like saturated fatty acids (SFAs) [25, 25], polyunsaturated fatty acids (PUFAs), and iron; non-dietary pro-oxidants such as smoking and obesity; non-dietary anti-oxidant such as physical activity; and dietary anti-oxidants for example fiber, vitamin C, vitamin B_9_, vitamin E, selenium, zinc, and beta-carotene [6, 8, 28–30]. Every score of these 13 components was summed, and the range of total score was between 0–26 (Table 1).

**Table 1:** Oxidative balance score components

OBS components	Assignment scheme
Non-dietary pro-oxidants	
Obesity	0 = BMI ≥ 30 kg/m^2^ and WC ≥ 1.02 m in males or ≥ 0.88 m in females
	1 = BMI ≥ 30 kg/m^2^ or WC ≥ 1.02 m in males or ≥ 0.88 m in females
	2 = BMI < 30 kg/m^2^ and WC < 1.02 m in males or < 0.88 m in females
Smoking	0 = current, 1 = former and 2 = never
Non-dietary anti-oxidants	
Physical activity (MET-min/d)	0 = low (1^st^ tertile), 1 = medium (2^nd^ tertile), and 2 = high (last tertile)
Dietary pro-oxidants	
SFA (g)	0 = high (3^rd^ tertile), 1 = medium (2^nd^ tertile), and 2 = low (1^st^ tertile)
PUFA (g)	0 = high (3^rd^ tertile), 1 = medium (2^nd^ tertile), and 2 = low (1^st^ tertile)
Iron (mg)	0 = high (3^rd^ tertile), 1 = medium (2^nd^ tertile), and 2 = low (1^st^ tertile)
Dietary anti-oxidants	
Fiber (g)	0 = low (1^st^ tertile), 1 = medium (2^nd^ tertile), and 2 = high (last tertile)
Vitamin E (mg)	0 = low (1^st^ tertile), 1 = medium (2^nd^ tertile), and 2 = high (last tertile)
Vitamin C (mg)	0 = low (1^st^ tertile), 1 = medium (2^nd^ tertile), and 2 = high (last tertile)
Vitamin B_9_ (µg)	0 = low (1^st^ tertile), 1 = medium (2^nd^ tertile), and 2 = high (last tertile)
Beta-carotene (mcg)	0 = low (1^st^ tertile), 1 = medium (2^nd^ tertile), and 2 = high (last tertile)
Zinc (mg)	0 = low (1^st^ tertile), 1 = medium (2^nd^ tertile), and 2 = high (last tertile)
Selenium (mg)	0 = low (1^st^ tertile), 1 = medium (2^nd^ tertile), and 2 = high (last tertile)

*OBS* Oxidative balance score, *BMI* Body mass index, *WC* Waist circumference, *MET* Metabolic equivalent of task, *SFA* Saturated fatty acid, *PUFA* Polyunsaturated fatty acid

According to a method developed by McCarty [17], the DPI was calculated by this formula: [DPI = (phytochemical-rich foods / total food intake) × 100]. Foods included fruits, vegetables, legumes, whole grains, soy products, nuts, olives, olive oil, spices, tea, and coffee. Natural fruit and vegetable juices were included in the fruit and vegetable groups, respectively, due to their phytochemical content.

### Socio-demographic and anthropometric assessments

Some information like physical activity, socio-demographic specifications, history of having CRC in their families, smoking habits, and medication use were collected by questionnaires. Anthropometric indices such as weight and height were measured. The international physical activity questionnaires (IPAQ) were utilized to evaluate the physical activity level [31].

### Statistical analysis

SPSS (version 23.0) was utilized for statistical analysis. The normality of data was determined by the Kolmogorov–Smirnov test. Mean (SD) or median (interquartile range (IQR)) was used for continuous variables, and the percentage was used for categorical variables. For categorical variables, the chi-square test was used; for continuous variables, independent samples T-test and Mann–Whitney were applied. Crude and adjusted models of logistic regression were utilized to assess the tertiles of OBS and DPI. The level of statistical significance was tested with a *p*-value < 0.05. Also, R software (version 3.0.2) was used for all figures' depictions.

## Results

Table 2 shows the basic characteristic of the case and control groups. Based on this table, OBS, DPI, vegetables, fruits, SFA, PUFA, fiber, vitamin E, vitamin C, beta-carotene, history of CRC, taking aspirin, and acetaminophen were significantly different between the case and control groups.

**Table 2:** Basic characteristic of the control and case groups

Variables	Cases (*n* = 71)	Controls (*n* = 142)	*P*-value
Age (year)^a^	58.2 ± 10.4	57.7 ± 10.4	0.746
BMI (kg/m^2^)^a^	27.6 ± 4.2	26.6 ± 4.2	0.362
Waist circumference (cm)^a^	95.4 ± 11.2	95.4 ± 11.9	0.904
Physical activity (MET-h/day)^a^	36.8 ± 3.6	36.7 ± 4.8	0.932
Income (dollar)^b^	393.0 (253.0)	402.0 (302.0)	0.206
Energy (kcal/day)^a^	2262.3 ± 450.1	2255.2 ± 341.2	0.908
Total OBS^b^	12.0 (3.0)	14.0 (3.0)	**<0.001**
DPI (energy %)^b^	19.8 (16.0)	26.9 (17.0)	**0.006**
Whole grains (kcal/day)^b^	120.4 (218.8)	180.9 (274.1)	0.100
Nuts (kcal/day)^b^	12.4 (21.2)	18.0 (32.6)	0.122
Legumes (kcal/day)^b^	39.6 (38.8)	40.3 (43.9)	0.745
Seeds (kcal/day)^b^	13.3 (18.3)	12.1 (18.3)	0.376
Vegetables (kcal/day)^b^	66.6 (41.6)	81.0 (47.5)	**0.001**
Fruits (kcal/day)^b^	128.7 (107.5)	160.4 (154.0)	**0.015**
SFA (g/day)^b^	18.3 (4.3)	28.6 (6.6)	**0.033**
PUFA (g/day)^b^	13.4 (1.2)	9.9 (1.9)	**<0.001**
Iron (mg/day)^b^	18.1 (5.2)	17.1 (5.4)	0.143
Fiber (g/day)^a^	18.9 ± 2.3	20.4 ± 3.1	**<0.001**
Vitamin E (mg/day/day)^b^	14.6 (1.3)	12.0 (1.8)	**<0.001**
Vitamin C (mg/day/day)^b^	104.0 (28.8)	132.7 (27.1)	**<0.001**
Vitamin B_9_ (µg/day)^a^	480.1 ± 115.0	482.6 ± 93.1	0.863
Beta-carotene (mcg/day)^b^	1099.0 (300.0)	1032.0 (434.1)	**0.012**
Zinc (mg/day)^a^	9.8 ± 2.2	10.1 ± 1.8	0.278
Selenium (mg/day)^b^	56.0 (3.0)	56.0 (3.0)	0.711
Smoking^c^			0.164
Never	57 (80.2)	101 (70.1)	
Former	8 (11.3)	15 (10.6)	
Current	6 (8.5)	26 (18.3)	
Common ways of preparing vegetables^c^			0.083
Raw / Fresh	29 (40.8)	78 (54.9)	
Boiled	8 (11.3)	18 (12.7)	
Fried, Fried / Freezed	34 (47.9)	46 (32.4)	
History of CRC^c^			**0.017**
Yes	7 (9.9)	3 (2.1)	
No	64 (90.1)	139 (97.9)	
Aspirin^c^			**0.016**
Yes	1 (1.4)	14 (9.9)	
No	70 (98.6)	128 (90.1)	
Acetaminophen^c^			**0.004**
Yes	4 (5.6)	28 (19.7)	
No	67 (94.4)	114 (80.3)	

Values are mean ± SD for continuous and percentage for categorical variables

*BMI* Body mass index, *OBS* Oxidative balance score, *MET* Metabolic equivalent of task, *DPI* Dietary phytochemical index, *SFA* Saturated fatty acids, *PUFA* Polyunsaturated fatty acids, *CRC* Colorectal cancer

^a^Using independent samples T-test for normal continuous variables

^b^Using Mann–Whitney for abnormal continuous variables

^c^Using chi-square test for categorical variables

**Fig. 2:**
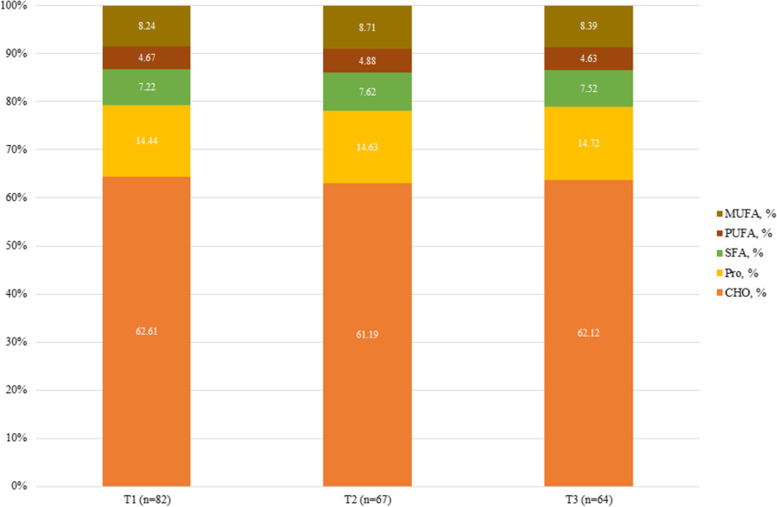
The contribution of macronutrient intake based on OBS tertile

**Fig. 3:**
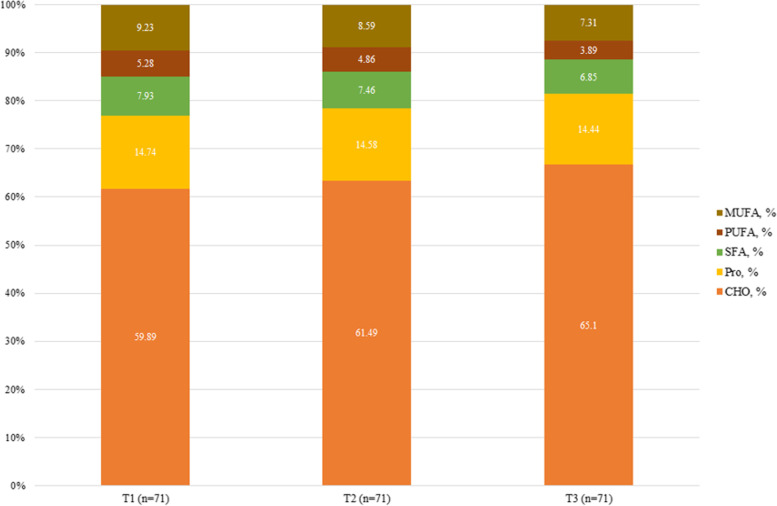
The contribution of macronutrient intake based on DPI tertile

**Fig. 4:**
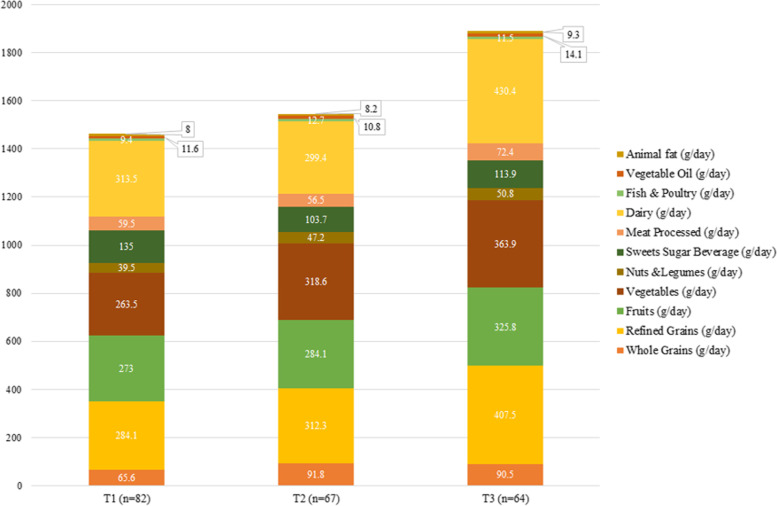
The consumption of food groups based on OBS tertile

**Fig. 5:**
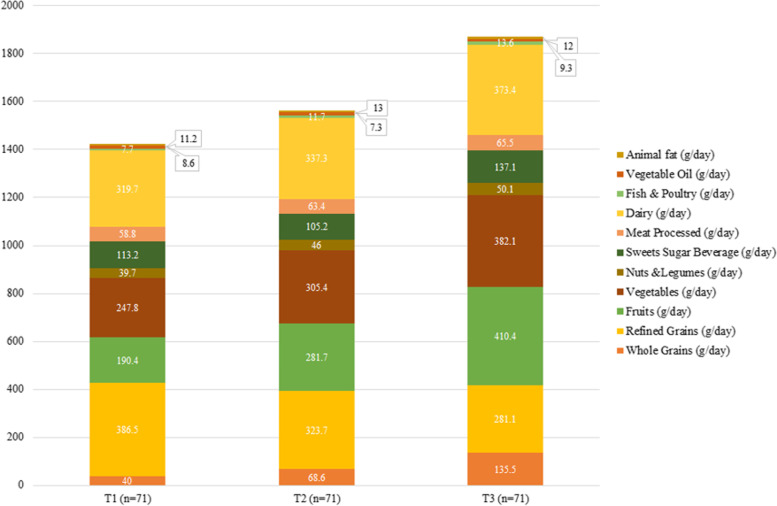
The consumption of food groups based on DPI tertile

Macronutrient and food intake across the tertiles of OBS and DPI are shown in Figs. 2, 3, 4 and 5. According to Fig. 2, participants in the last tertile of OBS had a higher significant intake of carbohydrates, SFA, monounsaturated fatty acids (MUFAs), and PUFA compared to the first tertile (*P*˂0.001 for all). But, according to Fig. 3, macronutrient intake was not significant between DPI tertiles. Compared to the first tertile, participants in the last tertile of OBS had higher significant consumption of refined grains (*P*˂0.001), vegetables (*P*˂0.001), processed meats (*P* = 0.008), dairy (*P*˂0.001), and vegetable oils (*P* = 0.01) (Fig. 4). Also, participants in the last tertile of DPI had higher consumption of whole (*P*˂0.001) and refined grains (*P* = 0.001), fruits (*P*˂0.001), vegetables (*P*˂0.001), fish, and poultry (*P* = 0.003), in comparison to the first tertile (Fig. 5).

In multivariate analysis (Table 3), the OBS was associated with a 77% lower odds for CRC in the last tertile than the first tertile (P_trend_ = 0.017). Furthermore, we detected a 64% significant reduction in the odds of CRC in the last tertile of DPI compared to the first tertile (P_trend_ = 0.015).

**Table 3:** Crude and multivariable-adjusted odds ratios and 95% CIs across tertiles of OBS and DPI

Variables	Case/Control	Crude Model	Adjusted Model
OBS			
T_1_ (≤ 12)	38/44	Ref	Ref
T_2_ (13–14)	24/43	0.64 (0.33–1.25)	0.60 (0.26–1.38)
T_3_ (≥ 15)	9/54	**0.19 (0.08–0.44)**	**0.23 (0.07–0.72)**
P_trend_		**< 0.001**	**0.017**
DPI			
T_1_ (≤ 19% energy)	33/38	Ref	Ref
T_2_ (20–30% energy)	21/50	**0.48 (0.24–0.96)**	0.48 (0.21–1.08)
T_3_ (≥ 31% energy)	17/54	**0.36 (0.17–0.74)**	**0.36 (0.15–0.86)**
P_trend_		**0.005**	**0.015**

Adjusted model: adjusted for energy, smoking, physical activity, Common methods of consuming vegetables, history of CRC, fiber intake, and education level

These values are odds ratio (95% CIs)

Obtained from logistic regression

*OBS* Oxidative balance score, *DPI* Dietary phytochemical index

## Discussion

The present study showed that OBS and DPI were associated with a significant reduction in CRC odds.

In agreement with our findings, a cohort study with a 10-year follow-up among 80,063 Americans and the Iowa Women's Health Study involving 33,736 women aged 55 to 69 years revealed an inverse association between OBS and the risk of colon cancer [6, 32]. Furthermore, two observational studies conducted in the United States (US) reported a negative association between OBS and colorectal adenoma risk [11, 12]. These studies were conducted in the US, which cannot be generalized to other populations due to dietary and environmental differences. The present study is the first study conducted in the Middle East. Anti-oxidants are divergent in terms of their chemical structure and biological characteristics. Anti-oxidants can inhibit proliferation, induce apoptosis and regulate the nuclear factor kappa B (NF-κB) and mitogen-activated protein kinase (MAPK) pathways, which can lead to cell death in cancer cells [33].

Our results demonstrated that higher DPI scores were significantly associated with lower CRC odds. To our knowledge, no previous study has assessed the association between DPI score and CRC. Regarding chronic disease, meta-analyses have revealed that higher adherence to DPI is associated with a lower risk of overweight/obesity [34], hypertension, hypertriglyceridemia, and metabolic syndrome [35]. In our study, participants in the highest DPI had a higher intake of whole grains, fruits, vegetables, fish, and poultry and a lower intake of refined grains than the lowest tertile. It indicates that their diet has more phytochemical content and anti-oxidant properties to act against cancer. Phytochemicals have been shown to modulate these pathways by neutralizing oxidative stress, inhibiting the NF-kB pathway, suppressing MAPK/extracellular signal regulated-kinases (ERK), and increasing the activation of caspase 3 [36].

According to World Health Organization (WHO), one-third to half of the cancer deaths could be prevented by lifestyle modification, including diet, weight management, exercise, and avoiding tobacco and alcohol consumption [37]. Diet has a bidirectional effect on the development of CRC [38]. A high intake of fruits, vegetables, whole grains, and dairy products, along with a low intake of red and processed meats, was associated with reduced CRC risk [39]. Also, a review study has shown that following a healthy pattern, represented by a high intake of whole grains, vegetables, fruits, legumes, nuts, seafood, and dairy products, reduces the risk of CRC [40]. Furthermore, an inverse association has been found between adherence to plant-based diet index (PDI) and CRC [41]. Dietary factors that have been proposed for their anti-cancer properties involve phytochemicals (polyphenols, flavonoids, alkaloids, etc.) [42], anti-oxidants (beta-carotene), probiotics, omega-3, and vitamins [43]. It has been found that cell cycle, apoptosis, and signaling pathways are altered in various types of cancer.

The present study has several strengths. Several potential confounders were included in the analysis to reach an independent association. A valid and reliable FFQ was used for measuring dietary intakes. Also, there were several limitations in the study. First, the case–control study cannot show a causal relationship between the variables. Second, FFQ is based on memory and a subjective approach to evaluating diet, which may lead to measurement errors.

## Conclusions

In conclusion, following a diet rich in phytochemicals and anti-oxidants, including fruits and vegetables (citrus fruits, colored berries, and dark-green leafy vegetables), and whole grains, may reduce the CRC odds. Further prospective studies and randomized clinical trials are warranted to confirm the association between DPI and OBS with CRC. It is also suggested that future studies consider the effects of a diet rich in anti-oxidants on the endogenous oxidative stress pathway in CRC.

## Supplementary Information

**Additional file 1.**

## Data Availability

The datasets used and/or analyzed during the current study are available from the corresponding author on reasonable request.

## Abbreviations

CRC: Colorectal cancer
DPI: Dietary phytochemical index
OBS: Oxidative balance score
FFQ: Food frequency questionnaire
DNA: Deoxyribonucleic acid
SFA: Saturated fatty acids
MUFA: Monounsaturated fatty acids
PUFA: Polyunsaturated fatty acids
IPAQ: International physical activity questionnaires
IQR: Interquartile range
NF-κB: Nuclear factor kappa B
MAPK: Mitogen-activated protein kinase
PDI: Plant-based diet index
ERK: Extracellular signal regulated-kinases
WHO: World health organization
